# Assessment of oligoclonal bands in cerebrospinal fluid and serum of dogs with meningoencephalitis of unknown origin

**DOI:** 10.1371/journal.pone.0280864

**Published:** 2023-01-25

**Authors:** Julia K. Prümmer, Veronika M. Stein, Eliane Marti, Andreas Lutterotti, Ilijas Jelcic, Gertraud Schüpbach-Regula, Thorsten Buch, Arianna Maiolini

**Affiliations:** 1 Division of Clinical Neurology, Vetsuisse Faculty, University of Bern, Bern, Switzerland; 2 Division of Neurological Sciences, Vetsuisse Faculty, University of Bern, Bern, Switzerland; 3 Department of Neurology, University of Zurich, Zurich, Switzerland; 4 Department of Clinical Research and Public Health, Vetsuisse Faculty, University of Bern, Bern, Switzerland; 5 Institute of Laboratory Animal Science, University of Zurich, Zurich, Switzerland; Policlinico Riuniti of Foggia: Neuroscience Department, S.C. Ospedaliera of Neurology-Stroke Unit, ITALY

## Abstract

**Background:**

Meningoencephalitis of unknown origin (MUO) is an inflammatory disease of the canine central nervous system (CNS) that shares several features with multiple sclerosis (MS) in humans. In approximately 95% of MS patients, ≥ two immunoglobulin G (IgG) oligoclonal bands (OCBs) are detectable exclusively in the cerebrospinal fluid (CSF).

**Hypothesis/objectives:**

To investigate OCBs in CSF and serum in dogs affected by MUO, intervertebral disc disease (IVDD), idiopathic epilepsy (IE), intracranial neoplasia (IN), steroid-responsive meningitis-arteritis (SRMA), and diseases outside the CNS. We hypothesize that the highest prevalence of CSF-specific OCBs (≥ two OCBs uniquely in the CSF) would be found in dogs affected by MUO.

**Animals:**

Client-owned dogs (n = 121) presented to the neurology service due to neurological deficits.

**Methods:**

Prospective study. Measurement of IgG concentration in CSF and serum via a canine IgG ELISA kit. OCB detection via isoelectric focusing (IEF) and immunoblot.

**Results:**

Presence of CSF-specific OCBs was significantly higher in dogs with MUO (57%) compared to 22% in IN, 6% in IE, 15% in SRMA, 13% in IVDD, and 0% in the non-CNS group (p < .001). Dogs with MUO were 9.9 times more likely to show CSF-specific OCBs than all other diseases together (95% confidence interval, 3.7–26.4; p < .001).

**Conclusions and clinical importance:**

MUO showed the highest prevalence of CSF-specific OCBs, indicating an inflammatory B cell response. Future studies are needed to evaluate the prevalence in the specific MUO subtypes and a possible similarity with human MS.

## Introduction

Meningoencephalitis of unknown origin (MUO) encompasses idiopathic, non-infectious inflammatory diseases of the canine central nervous system (CNS), which can be further subdivided into granulomatous meningoencephalitis (GME), necrotizing meningoencephalitis (NME), and necrotizing leukoencephalitis (NLE) via histopathological examination [1]. Although the exact number of affected dogs is currently not known and may vary among countries, early studies reported an incidence of GME of up to 25% of all canine CNS disorders [2]. Attempts to identify an underlying infectious agent have failed so far. Thus, an autoimmune pathogenesis is suspected [3–6]. Major factors contributing to the disease are genetic susceptibility and environmental factors [1].

Studies have highlighted characteristics [7–10] similar to multiple sclerosis (MS), an inflammatory demyelinating disease affecting the CNS of humans [11]. For instance, a strong dog leukocyte antigen (DLA) class II association in Pug Dogs affected by NME has been found, resembling the association between human leukocyte antigen (HLA) class II and MS in human patients [7]. Moreover, a gender predisposition for females has been recognized in both diseases [9, 10]. A comparison between the canine MUO and MS in humans can be found in Table 1. The diagnostic guidelines for MS are summarized in the McDonald criteria from the International Panel of MS [12]. In these criteria, immunoglobulin G (IgG) oligoclonal bands (OCBs) remain a valuable diagnostic test, as evidence of intrathecal antibody synthesis supports the diagnosis. However, those bands are not specific for MS, which is why clinical, imaging as well as laboratory evidence needs to be interpreted together to reach a diagnosis of MS [12]. CSF abnormality is defined by the presence of OCBs different from any such bands in serum. These bands are found in CSF in chronic inflammatory diseases of the CNS due to increased local antibody synthesis. The method most widely used for their detection is isoelectric focusing (IEF) on agarose gel followed by immunoblotting or immunofixation for IgG using paired CSF and serum samples [13–15]. A previous meta-analysis reported the diagnostic sensitivity of CSF OCBs for MS using the above-mentioned gold standard to be 93%, respectively [16].

**Table 1:** Comparison of MS in humans and canine MUO.

Parameters for comparison	MS	MUO
**Signalement** [1, 17]	Young adults Female:male 3:1	3–7 years Small, female dogs
**Genetic factors**	HLA-DRBI*15 [18]	NME [7] NME-susceptibility haplotype close to the DLA complex
**Clinical presentation** [1, 11]	Spinal cord syndrome Optic neuritis Brainstem or cerebellar syndrome Cognitive impairment Others	GME Multifocal (forebrain, brainstem, spinal cord) Focal Ocular NME Forebrain NLE Forebrain and brainstem
**Course** [1, 17]	Variable: Relapsing-remitting Primary progressive Secondary progressive Progressive relapsing	Acute onset Rapid progression
**CSF pleocytosis** [1, 19]	Up to 50 cells/μl Mononuclear: predominantly lymphocytes, some monocytes	Generally mononuclear. GME Mild to moderate lymphocytic, neutrophilic, mixed NME, NLE Moderate to marked lymphocytic
**CSF-specific OCBs** [19]	Present 90%–> 95%	Not investigated before
**MRI findings** [1, 17]	Ovoid, well circumscribed Periventricular, juxtacortical, infratentorial, spinal cord Hyperintensity on T2W Contrast enhancement of new lesions for approximately 6 weeks	GME Multifocal, diffuse Hyperintensity on T2W and FLAIR Variable T1W contrast enhancement NME Asymmetric, multifocal cerebrocortical gray and white matter lesions Hyperintensivity on T2W and FLAIR Variable T1W contrast enhancement Meningeal enhancement NLE Asymmetric cerebral white matter and brainstem lesions Hyperintensivity on T2W and FLAIR Minimal contrast enhancement
**T vs B cell involvement**	B- and T-cell involvement [20]	GME [3] CD3^+^ T cells CD163^+^ macrophages Mainly T cells identified, B cells rarely seen

MUO = meningoencephalitis of unknown origin, MS = multiple sclerosis, HLA = human leukocyte antigen, DLA = dog leukocyte antigen, NME = necrotizing meningoencephalitis, GME = granulomatous meningoencephalitis, NLE = necrotizing leukoencephalitis, CSF = cerebrospinal fluid, OCB = oligoclonal band, MRI = magnetic resonance imaging

Similar to MS, the pathogenesis of MUO is not fully understood. So far, a T cell-mediated delayed-type hypersensitivity with involvement of mainly T and just few B cells has been postulated in dogs with GME [3]. The lack of knowledge about the pathogenesis is reflected by the absence of specific diagnostic tests for ante mortem definitive diagnosis. The combination of clinical signs, imaging features, CSF findings, and testing for infectious diseases helps to reach a presumptive diagnosis: magnetic resonance imaging (MRI) has a good diagnostic value with a sensitivity of 86% and specificity of 93.1% for classifying brain disease as inflammatory [21]. Accordingly, a normal brain MRI does not rule out inflammatory diseases of the CNS [22] and canine brain granuloma can be misdiagnosed as glioma [23]. Therefore, histopathology remains the gold standard, especially for diagnosing the MUO subtypes. The presence of CSF-specific OCBs in dogs affected by MUO may help to distinguish between inflammatory and neoplastic disorders, to non-invasively differentiate the MUO subtypes, and to highlight further similarities between the canine MUO and MS in humans.

The only clinical study available in veterinary medicine on OCBs in German Shepherd dogs diagnosed with degenerative myelopathy involved just a small number of diseased and healthy dogs and lacked the comparison with other neurological diseases [24].

The aim of the present study therefore was to investigate CSF and serum for the presence of CSF-specific OCBs with IEF and immunoblot in dogs affected by MUO in comparison to dogs affected by idiopathic epilepsy, intracranial neoplasia, intervertebral disc disease, steroid-responsive meningitis-arteritis, or diseases not affecting the CNS. We hypothesized that the highest prevalence of CSF-specific OCBs would be found in dogs affected by MUO.

## Materials and methods

The experimental procedures used in this study were approved by the ethical committee of the Veterinary service, Cantone of Bern (BE121/2020).

Sampling of CSF and serum was done during the diagnostic work-up of each patient (no sampling was done only for research purposes), and all efforts were made to minimize stress and suffering for each patient. In addition, we have obtained the owner’s written consent for each of the dogs involved in this study. Human samples were taken during the diagnostic work-up of the patient in the University Hospital, Zurich (not taken only for the purpose of the study). Patients signed a consent that residual amount of CSF and serum not needed for the diagnostic work-up can be used for research purposes. Samples were anonymized prior to researchers having access to the samples.

CSF and serum samples were prospectively collected from dogs presented to the Small Animal Clinic, Division of Clinical Neurology, Vetsuisse Faculty, University of Bern, Switzerland due to neurological impairment between April 2018 and June 2020. Patients were included in the study if all criteria were met: All dogs had a complete medical record including signalment, they received a complete diagnostic work-up with clinical and neurological examination, full blood work (hematology, biochemistry, infectious disease testing if indicated), MRI of the brain and/or spinal cord and CSF puncture. When possible, dogs received a histopathological and immunohistopathological examination of brain biopsies/the brain.

Dogs were classified into six disease categories: meningoencephalitis of unknown origin (MUO), idiopathic epilepsy (IE), intracranial neoplasia (IN), intervertebral disc disease (IVDD), steroid-responsive meningitis-arteritis (SRMA), and non-CNS category. A clinical diagnosis of MUO was based on the following criteria: (1) dogs older than 6 months, (2) multiple, single or diffuse intra-axial hyperintensities on T2-weighted MR images, and/or (3) pleocytosis in CSF analysis with > 50% of mononuclear cells, and (4) exclusion of infectious diseases (adapted from Granger et al. 2010 [25]). Response to treatment was additionally taken into account to support the diagnosis (follow-up of at least 11 months).

Dogs were assigned to the IE category, if they presented recurrent epileptic seizures and their diagnostic work-up met the Tier 2 confidence level as defined by the International Veterinary Epilepsy Task Force (IVETF) [26]. Dogs were included in the IN category, if they were diagnosed with a neoplastic lesion based on MRI and subsequent CSF examination, ideally confirmed by histopathological examination. Dogs were diagnosed with IVDD based on MRI examination and confirmed by surgery if indicated. Dogs were diagnosed with SRMA based on clinical signs, CSF findings (neutrophilic pleocytosis) [27] and response to treatment. Dogs were allocated to the non-CNS disease category, if diseases were located outside the CNS with a normal MRI examination of the brain as well as an unremarkable CSF examination (e.g. dogs with otitis media).

Paired serum and CSF samples were collected from all dogs during the diagnostic work-up. CSF was obtained by atlanto-occipital puncture in lateral recumbency during general anesthesia and examined within 30 minutes for total protein and total nucleated cell count (TNCC) as well as cytology. Pleocytosis was defined as a TNCC > 5 cells/μL and was further characterized as mild (6–50 cells/μL), moderate (51–200 cells/μL) or marked (> 200 cells/μL) [28]. The pleocytosis was further characterized by the predominant cell type as neutrophilic, mononuclear, eosinophilic, or mixed without a clear predominance of one cell type [28]. Protein content was measured using the benzethonium chloride method (Cobas c501, Total Protein Urine/CSF Gen. 3, Roche) and defined as normal if < .33 g/L and increased if > .33 g/L. Blood samples mostly were taken at the same time or within 24 hours by puncture of a peripheral vein (e.g. V. saphena lateralis or V. cephalica antebrachii). Serum samples were centrifuged and both CSF and serum samples frozen in plain plastic tubes at -80°C within one hour from sampling. Samples were directly thawed prior to use. Measurements of IgG concentrations in both CSF and serum samples were performed in the laboratory of Clinical Immunology of the Vetsuisse Faculty in Bern using a Canine IgG ELISA kit (Abcam, Cambridge, United Kingdom), following the manufacturer’s instructions. After determination of IgG concentrations, samples were aliquoted and again frozen at -80°C until analyzing the presence of OCBs in the "CSF Laboratory", Department of Neurology, University Hospital Zurich, via IEF followed by immunoblotting (SEBIA Swiss GmbH, Wollerau, Switzerland) as previously described [29]. Samples were diluted with Aqua ad iniectabilia to reach equal IgG concentrations of 25mg/l in CSF and serum prior to analysis. Proteins were separated via the IEF and then blotted onto a membrane via immunoblot. OCBs were then highlighted via the use of a canine rabbit-anti-IgG antibody (Jackson ImmunoResearch Laboratories, Ely, United Kingdom; H+L, purified, polyclonal, Alkaline Phosphatase conjugated; product code 304-055-033). The presence of OCBs was independently evaluated by three blinded experienced raters (AL, MZ, EdA). If only two out of three agreed on the result, the most experienced examiner (AL) reevaluated the blots to reach a consensus. Similar to the guidelines used for OCB detection in CSF and serum in human medicine, dogs were considered to have CSF-specific OCBs, i.e. intrathecal IgG synthesis, if two or more OCBs were detectable in CSF, but absent in corresponding serum, equivalent to OCBs type 2 (normal serum and additional OCBs in CSF) and OCBs type 3 (identical OCBs in serum and CSF with additional bands in CSF not present in serum) [13, 15, 30]. Type 1 reflects no OCBs in serum or CSF, type 4 identical OCBs in serum and CSF, reflective of a systemic, but not intrathecal IgG synthesis, and type 5 the presence of identical monoclonal bands in CSF and serum [13, 15]. Due to the lack of canine controls with confirmed CSF-specific OCBs, human CSF and serum samples with confirmed CSF-specific OCBs were used as positive control on all blots.

### Statistical analysis

Statistical analysis was performed using NCSS (NCSS 12 Statistical Software; LLC, Kaysville, Utah, United States). For all analyses, p < .05 was considered statistically significant. Summary descriptive statistics were calculated to describe the population of patients included as well as different CSF findings and pathology results. Concentrations were non-normal distributed. Therefore, the median was used additionally to describe total protein, TNCC, IgG in CSF and serum per disease category. A Kruskal-Wallis test was used for assessment of group differences of continuous, non-parametric data (IgG concentration in CSF and CSF-specific OCBs, IgG concentration in CSF and TNCC). Moreover, associations were assessed between the presence of CSF-specific OCBs and the TNCC as well as CSF cytology. Logistic regression and contingency tables were used to evaluate a possible relation between CSF-specific OCBs and single disease categories, and MUO versus all other disease categories summarized. Kappa statistics were used to assess the interobserver agreement.

## Results

125 dogs were initially included in the study. Four dogs from the MUO category had to be excluded as they were diagnosed with idiopathic cerebellitis (n = 2) and eosinophilic meningoencephalitis (n = 2). Therefore, 121 dogs were enrolled in the present study comprising 28 dogs with MUO, 18 dogs with IE, 23 dogs with IN, 13 dogs with SRMA, 23 dogs with IVDD, and 16 dogs with non-CNS disease (Table 2). The most common breeds were French Bulldog (17/121), mixed breed (16/121), Chihuahua (9/121), and Dachshund (6/121). Forty-four percent of the dogs (53/121) were female (15/53 intact, 38/53 neutered) and 56% were male (29/68 intact, 39/68 neutered). The mean age of the study population was 5 years and 4 months (range: 2 months– 12 years and 7 months) and mean body weight was 16 kg (range: 1.11–56.7 kg).

**Table 2:** Numbers of dogs included per disease category as well as prevalence of CSF-specific OCBs (≥ 2 OCBs uniquely in CSF) per disease category.

Disease category	MUO	IE	IN	IVDD	SRMA	Non-CNS
Dogs n = 121	28	18	23	23	13	16
CSF-specific OCBs	16 (57%)	1 (6%)	5 (22%)	3 (13%)	2 (15%)	0 (0%)

MUO = meningoencephalitis of unknown origin, IE = idiopathic epilepsy, IN = intracranial neoplasia, IVDD = intervertebral disc disease, SRMA = steroid-responsive meningitis-arteritis, non-CNS = diseases outside the CNS, OCBs = oligoclonal bands, CSF = cerebrospinal fluid

In the MUO disease category, 15/28 dogs showed a good response to treatment and were still alive at the time of writing, 2/28 were lost to follow-up and 11/28 died or were euthanized. Of the latter, a postmortem examination was declined by the owner in 7/28 dogs. Histopathology was available in 4/28 dogs with MUO (GME n = 1, NME n = 1, mixed type n = 2) and 16/23 dogs with IN (oligodendroglioma WHO grade II/III n = 7, meningioma n = 2, astrocytoma, atypical meningioma, choroid plexus carcinoma, histiocytic sarcoma, meningoangiomatosis, metastatic carcinoma, metastatic round cell tumor, each n = 1).

Total protein in CSF was available in 97/121 dogs with a median of .2 g/L and a mean of .45 g/L (5th-95th percentile .1–1.90 g/L). The TNCC was determined in 119/121 dogs with a median of two cells/μL and a mean of 200 cells/μL (5th-95th percentile .3–1234.7 cells/μL). Total protein was not determined in cases with CSF examination in the emergency night shifts, when only Pandy reaction was assessed. TNCC was not recorded in one dog with IN and one dog with IVDD. As only three dogs were found with a moderate pleocytosis, the categories moderate pleocytosis and marked pleocytosis were grouped together as marked pleocytosis to simplify the statistical analysis. The IgG concentration in CSF and serum was measured in all samples with a median of 46.93 mg/L in CSF and 22.11 g/L in serum and a mean of 191.74 mg/L (5th-95th percentile 11.78–1080.64 mg/L) in CSF and 23 g/L (5th-95th percentile 10.43–38.24 g/L) in serum. Medians as well as range of total protein, TNCC, IgG in serum and CSF per disease category can be found in Table 3.

**Table 3:** Median values as well as range (in brackets) of total protein (TP), total nucleated cell count (TNCC), IgG in serum and CSF per disease category.

Disease category	TP^1^ (g/L)	TNCC^2^ (cells/μl)	IgG serum (g/L)	IgG CSF^3^ (mg/L)
MUO	.3 (.15–2.21)	17.00 (.3–3754.0)	25.54 (12.35–42.82)	224.92 (19.44–3128.31)
IE	.14 (.1- .28)	.7 (.3–3.7)	21.54 (4.82–33.41)	21.38 (8.8–75.3)
IN	.27 (.12–1.52)	2.7 (.3–43.7)	21.66 (4.0–38.25)	60.18 (18.33–287.44)
IVDD	.13 (.1- .35)	1.45 (.7–15.30)	21.72 (9.04–45.5)	36.5 (11.54–1127.55)
SRMA	.75 (.09–6.32)	880.0 (52.00–4490.70)	16.15 (10.09–28.17)	209.00 (8.54–1834.81)
non-CNS	.17 (.07- .28)	.7 (.3–2.7)	22.14 (12.82–44.57)	29.95 (9.71–70.58)

MUO = meningoencephalitis of unknown origin, IE = idiopathic epilepsy, IN = intracranial neoplasia, IVDD = intervertebral disc disease, SRMA = steroid-responsive meningitis-arteritis, non-CNS = diseases outside the CNS, CSF = cerebrospinal fluid.

^1,2^ p < 0.001,

^3^ p < 0.01

**Fig 1:**
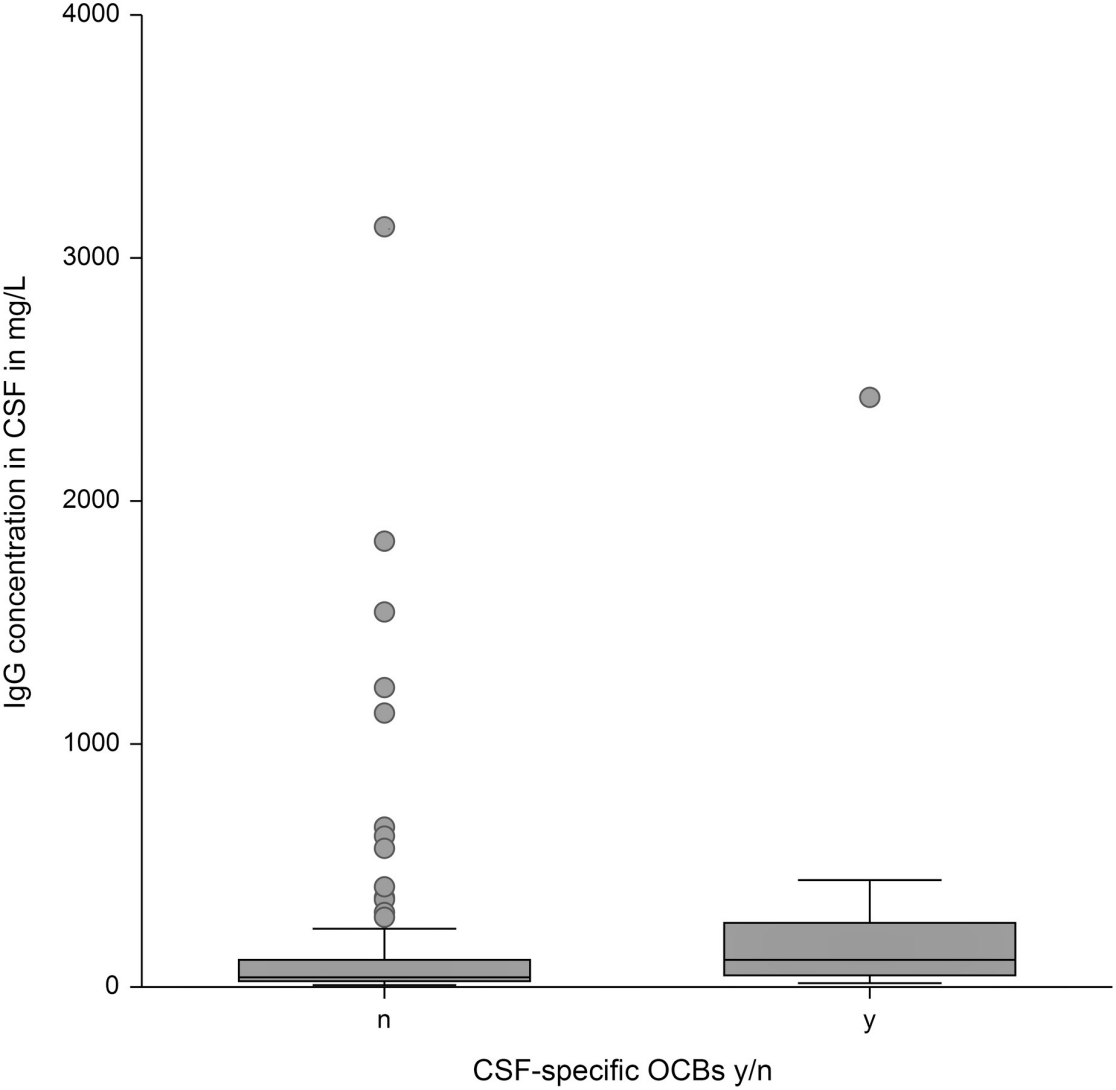
Association between IgG concentration in CSF and presence/absence of CSF-specific OCBs. An increased IgG concentration in CSF was significantly associated with the presence of CSF-specific OCBs (p < .01). IgG = immunoglobulin G, CSF = cerebrospinal fluid, OCB = oligoclonal band, y = yes, n = no.

**Fig 2:**
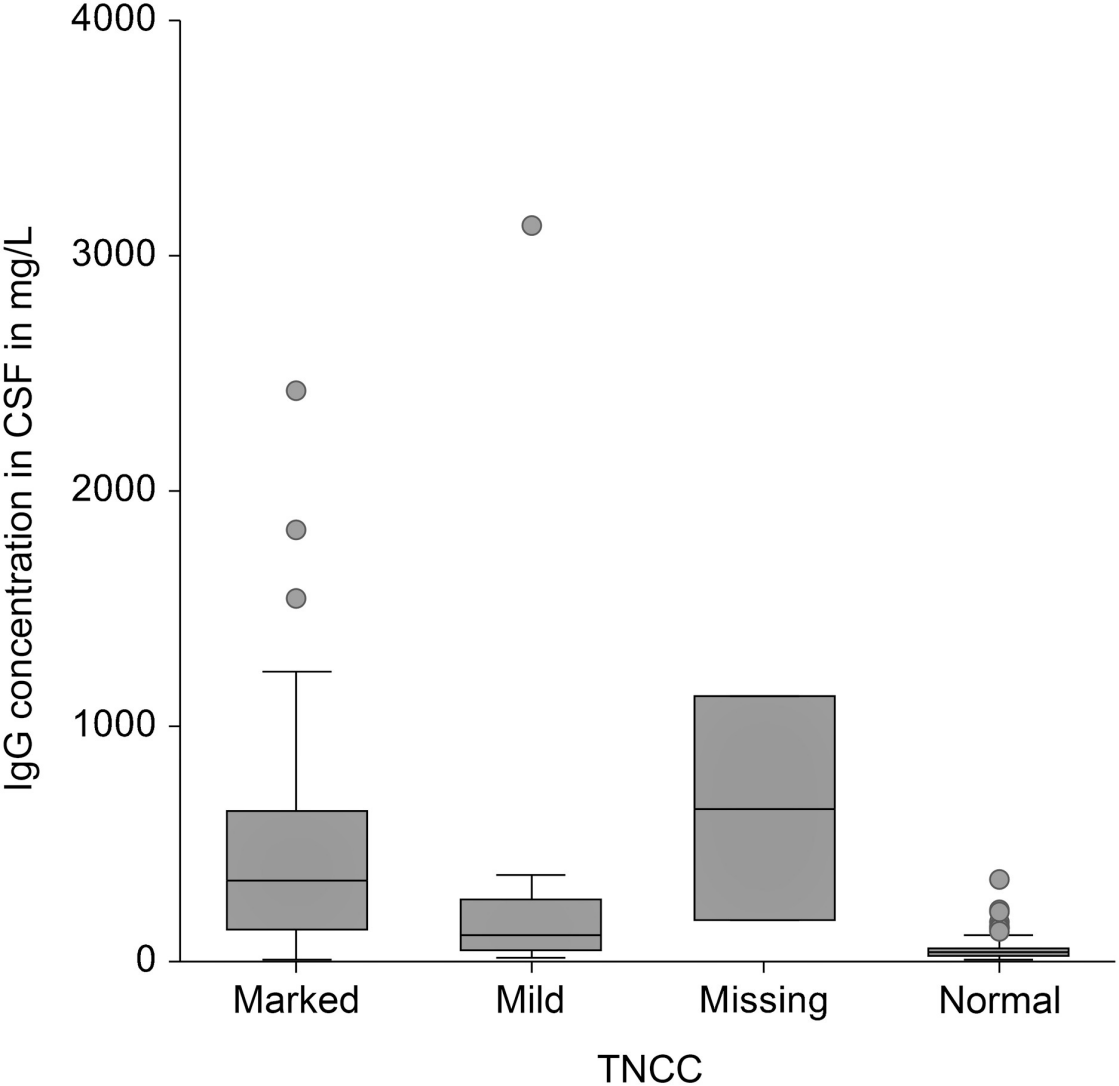
Association between IgG concentration and TNCC. An increased IgG concentration in CSF was significantly associated with an increased TNCC (p < .001). y = yes, n = no, CSF = cerebrospinal fluid, OCB = oligoclonal band, TNCC = total nucleated cell count.

**Fig 3:**
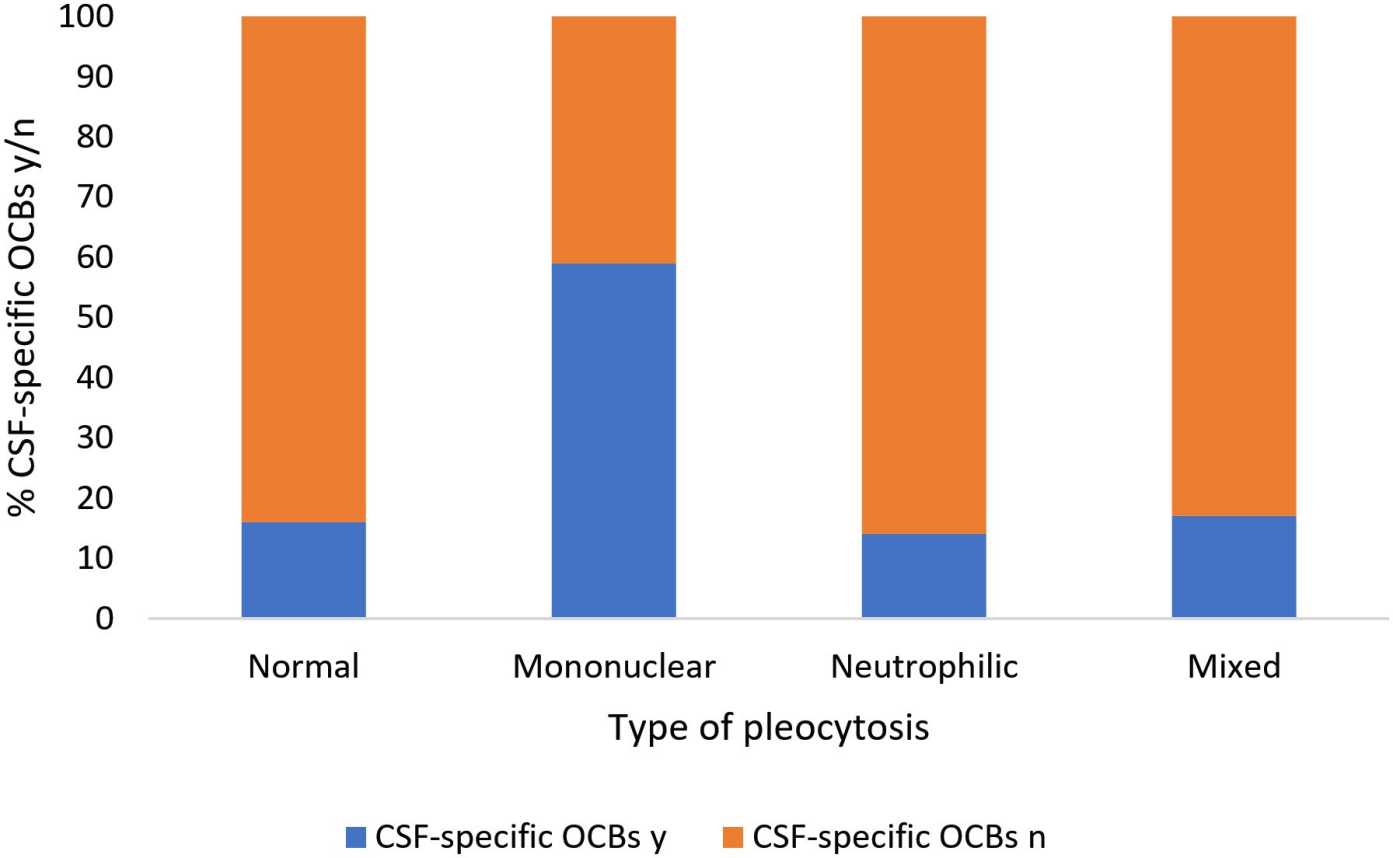
Association between presence/absence of CSF-specific OCBs and type of pleocytosis. Presence of CSF-specific OCBs was significantly associated with a mononuclear pleocytosis (p < .001). y = yes, n = no, CSF = cerebrospinal fluid, OCB = oligoclonal band.

**Fig 4:**
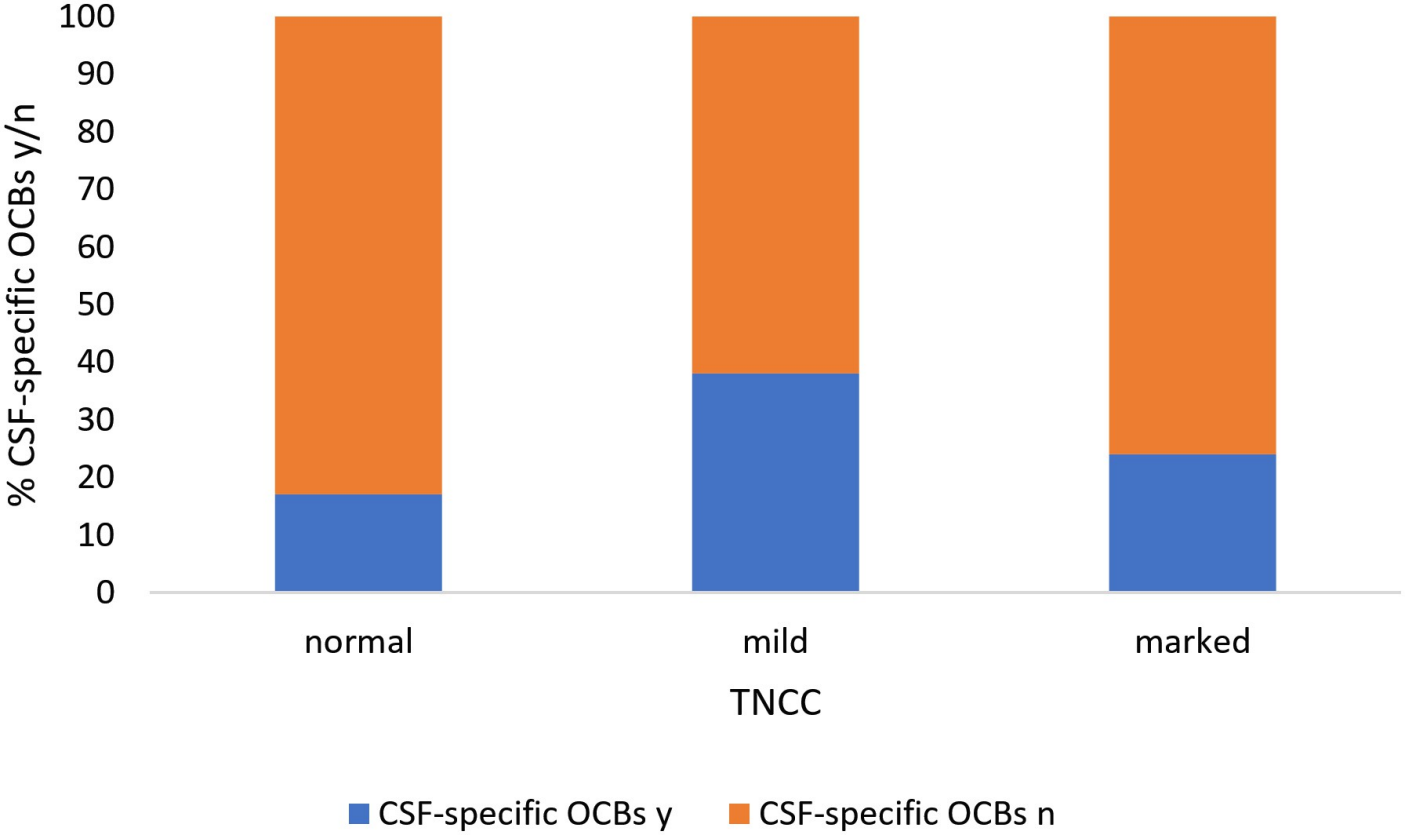
Association between presence of CSF-specific OCBs and TNCC. There was no association between presence of CSF-specific OCBs and increased TNCC present (p > .11). CSF = cerebrospinal fluid, OCB = oligoclonal band, y = yes, n = no, TNCC = total nucleated cell count.

An increased IgG concentration in CSF was significantly associated with the presence of CSF-specific OCBs (p < .01) (Fig 1) and an increased TNCC (p < .001) (Fig 2). Moreover, the presence of CSF-specific OCBs was significantly associated with a mononuclear pleocytosis (Fig 3). Fifty-nine percent of dogs with a mononuclear pleocytosis had CSF-specific OCBs compared to 16% with a normal cytology (p < .001). Presence of CSF-specific OCBs was not associated with an increased TNCC (Fig 4).

**Fig 5:**
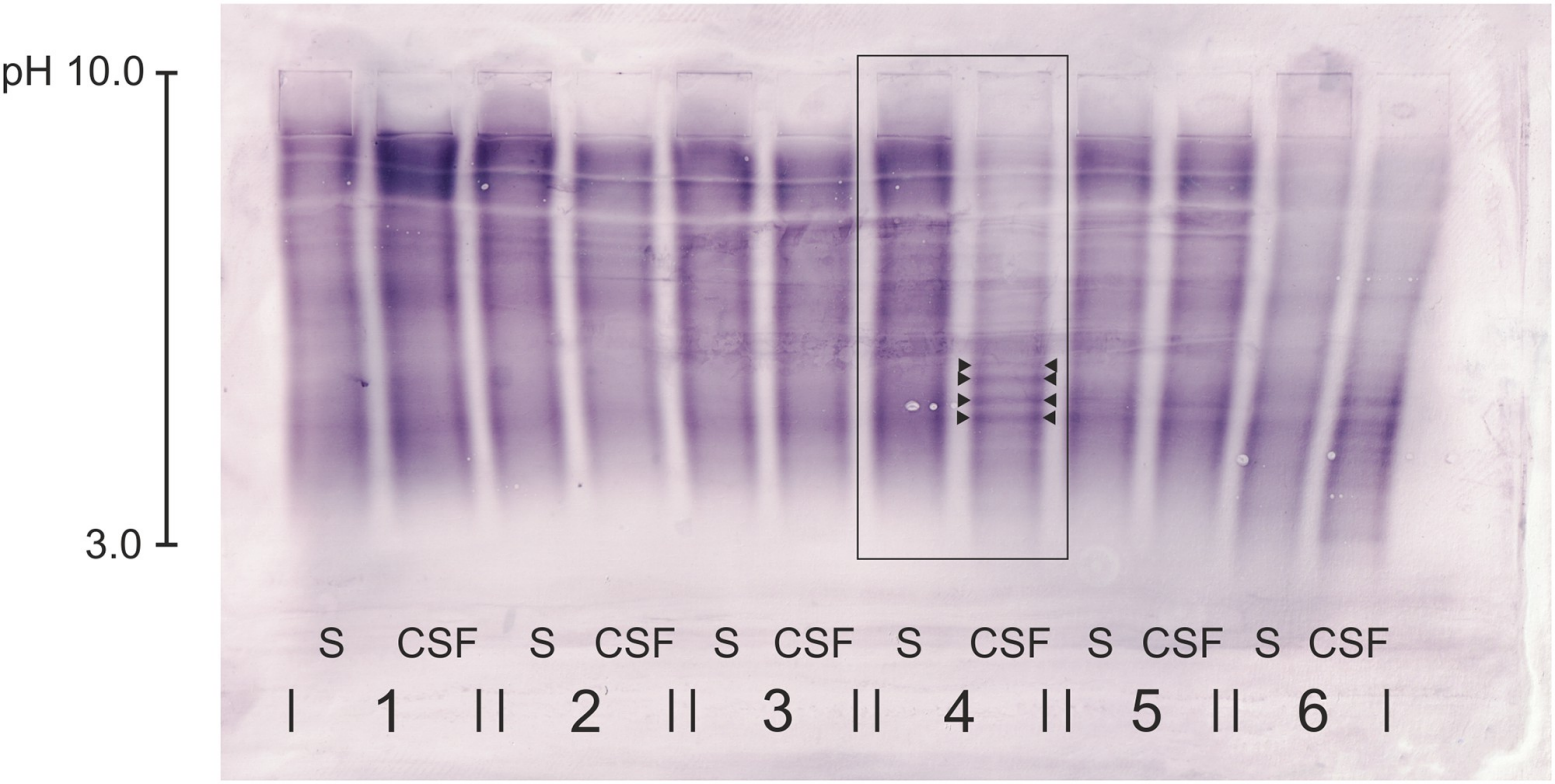
Immunoblot with serum and CSF samples from five canine patients (nos. 1–5) and one human MS patient (no. 6) after isoelectric focusing. Dog no. 1 was diagnosed with intracranial neoplasia, dog no. 2 and dog no. 3 with idiopathic epilepsy, dog no. 4 with MUO, and dog no. 5 with a disease outside the CNS. CSF-specific OCBs (equivalent to pattern 2 and 3 of the described patterns in human medicine; OCBs highlighted between arrowheads) were detected in dog no. 4. The samples from the human MS patient served as positive controls. CSF = cerebrospinal fluid, MS = multiple sclerosis, MUO = meningoencephalitis of unknown origin, CNS = central nervous system, OCBs = oligoclonal bands.

Presence of CSF-specific OCBs was significantly higher in dogs diagnosed with MUO (Fig 5) compared to every other disease category (p < .001). More specifically, 57% of dogs with MUO, compared to 22% of dogs with IN, 6% of dogs with IE, 15% of dogs with SRMA, 13% of dogs with IVDD, and 0% of dogs diagnosed with a disease not affecting the CNS were found to show CSF-specific OCBs (Table 2). When comparing the MUO category with all other disease categories summarized in one group, dogs with MUO were 9.9 times more likely to present CSF-specific OCBs than dogs with the other diseases (95% confidence interval, 3.7–26.4; p < .001). Agreement between the three observers was very good, with kappa values of .97 (95% CI .92–1) and .93 (95% CI .84–1), respectively.

## Discussion

The pathogenesis of human MS and the MUOs in canine patients are both not fully understood. Consequently, both diseases lack specific diagnostic tests for ante mortem definitive diagnosis. Instead, a range of defined clinical criteria is used to narrow down the diagnosis, one of which is the presence of CSF-specific OCBs as part of the McDonald criteria to diagnose MS. Up to 95% of human MS patients present with CSF-specific OCBs [13]. Similarities between human MS and canine MUO has already been postulated. However, OCBs have only been investigated in a group of dogs diagnosed with degenerative myelopathy so far [24]. Studies investigating the prevalence of CSF-specific OCBs in canine inflammatory diseases in the CNS and comparison with the described prevalence in human MS are lacking. Therefore, the present study aimed at investigating the prevalence of CSF-specific OCBs of dogs diagnosed with MUO compared to dogs with miscellaneous CNS diseases, as well as diseases not primarily affecting the CNS. The highest prevalence of CSF-specific OCBs was detected in the MUO disease category compared to all other disease categories. The prevalence in MUO is however much lower than the prevalence in human MS patients.

One potential explanation might be that the majority of dogs showed a good response to treatment and was therefore still alive at the time of writing or lost to follow-up resulting in a low number of patients with histopathological confirmation of MUO. In the majority of cases, the diagnosis of MUO was based on clinical factors, hence it cannot be excluded that neoplastic or other inflammatory diseases were included in the MUO disease category, lowering the actual prevalence of CSF-specific OCBs in the MUO category. The clinical diagnosis of MUO was based on information from clinical and neurological examination, full blood work (hematology, biochemistry, infectious disease testing), MRI of the brain and/or spinal cord and CSF puncture. There are currently no clear and internationally recognized diagnostic criteria defined for diagnosis of MUO. In the present study, the guidelines provided by Granger et al. presenting criteria for the diagnosis of MUO were followed [25], although also 6 dogs without CSF pleocytosis were included. According to these guidelines, CSF "should" be hypercellular, with > 50% mononuclear cells. Dogs without pleocytosis that otherwise fulfilled the remaining criteria therefore were not excluded, since normal CSF has been described in histopathologically confirmed MUO dogs: one study found 28.6% of cases with histologically confirmed NLE and 14.3% of Pug dogs with histologically confirmed NME to have a normal CSF [31]. Nevertheless, it cannot be entirely ruled out that dogs actually affected by other diseases were included in the MUO disease category. Moreover, the absence of OCBs in CSF and serum (pattern 1 of the previously mentioned IEF patterns) does not always exclude a CNS pathology, as it can occur in the very early stage during the disease course [16]. Mostly, dogs with MUO present with severe neurological signs and the owner might have not recognized mild initial deficits. Thus, it seems relatively unlikely that MUO patients present that early in the disease course that there is lack of an inflammatory response in the CNS. However, some patients present with less severe deficits, which might correlate with the mainly affected region of the brain and/or an early phase of the disease.

In human medicine, the overall prevalence of CSF-specific OCBs in inflammatory diseases in general, but not MS specifically, is not well established. However, the prevalence range is wide, with around 95% in MS, and for example 16.4% in neuromyelitis optica [32], 6–17% in myelin oligodendrocyte glycoprotein antibody-associated disease [33], 25% in Sjögren’s syndrome [34], and 29–58% in acute disseminated encephalomyelitis [35, 36]. Thus, the true prevalence of CSF-specific OCBs in MUO might be 57%; however, one of the MUO subtypes might present with a higher prevalence.

Each of the MUO subtypes presents with specific features, such as predilection for white matter in NLE and GME [1], or a genetic predisposition, more specifically a DLA class II association, in NME [7]. Those features resemble the presentation in human MS, with characteristic lesions within the white matter [12] and a major histocompatibility complex class II genetic association [37]. In the current study, a further differentiation in the MUO subtypes by histopathology was only available in 4/28 patients. This was due to good treatment response, loss to follow-up or decline of a postmortem examination by the owners. Of those, 3/4 dogs presented with CSF-specific OCBs (GME n = 1, mixed type n = 2). Due to the low number of cases, it was not possible to determine specific MUO subtypes in the majority of the study population and only the overall prevalence of CSF-specific OCBs in MUO and not a potential association between the subtypes GME, NME, and NLE, could be established.

Studies in human medicine demonstrated OCBs in various non-inflammatory neurological diseases with a prevalence of 67% in glioma patients, and 8.7–14% in patients with epileptic seizures [38, 39]. In the presented study population, the prevalence of CSF-specific OCBs ranged from 0–22% with 22%, 15%, 13%, 6%, and 0% in IN, SRMA, IVDH, IE, and the non-CNS disease category, respectively. Overall, 16/23 dogs (70%) in the IN category received a histopathological confirmation of the underlying disease, with oligodendroglioma being the most common diagnosis (8/16). Further tumor types were each represented just once. Due to the scattered distribution of tumor types, a correlation between presence of CSF-specific OCBs and type of neoplasia was not possible. Nevertheless, CSF-specific OCBs were present only in 2/8 (= 25%) of glioma patients, which is much less than the described prevalence of 67% in human glioma patients [38]. The latter prevalence derives from a study that included only a small number of patients and therefore these data might not reflect the real situation. Interestingly, the cases diagnosed with histiocytic sarcoma, atypical meningioma, and metastatic round cell tumor presented with CSF-specific OCBs.

Ante mortem differentiation between inflammatory and neoplastic lesions can be challenging and so far a definitive diagnosis can be reached only via biopsy [23, 40]. Using the OCB pattern for differentiation would have been an interesting, less invasive approach, however, with 22% of IN cases presenting CSF-specific OCBs, this seems not feasible. Although the majority of the IN cases in the current study underwent histopathological confirmation, a misdiagnosis in the remaining 30% of the cases is still a possibility that might have influenced the real prevalence of CSF-specific OCBs in this group.

SRMA is an inflammatory disease that does not solely affect the CNS but also has a strong systemic effect due to the widespread vasculitis. Therefore, the majority of cases was expected to show either a systemic OCB pattern (equivalent to type 4 [13]) or CSF-specific OCBs. On the contrary, identical OCBs in CSF and serum were not detected in any of the SRMA cases investigated (systemic pattern). This is corresponding to findings in human medicine with a less frequent association of type 4 with systemic autoimmune diseases [41]. The presence of CSF-specific OCBs in 2/15 cases in this group is not surprising, reflecting a secondary inflammatory process in those diseases as well as the fact that the presence of OCBs itself is unspecific and cannot be used as a diagnostic modality on its own as they can be found in various different diseases. Interestingly, CSF-specific OCBs were detected in each one of the two patients with eosinophilic meningoencephalitis and with idiopathic cerebellitis.

Finally, the prevalence of CSF-specific OCBs in dogs diagnosed with IE was 6% (1/18] and comparable with findings in human epileptic patients, in which a prevalence range of 8.7–14% is reported [39]. Interestingly, the only epileptic dog that presented CSF-specific OCBs was refractory to anticonvulsive treatment and finally euthanized. Unfortunately, the owner declined a postmortem examination. This dog showed changes in the MRI within the left piriform lobe, which were interpreted as postictal changes. According to the IVETF, repeated MRI of the brain with initially suspected postictal changes after a period of seizure control, along with clinical and CSF analysis findings may help to differentiate from structural epilepsy [26]. The presence of CSF-specific OCBs in this dog raised the question if the initial diagnosis was incorrect and the dog was actually affected by an MUO or if dogs diagnosed with IE that are refractory to treatment show CSF-specific OCBs and might actually have an underlying inflammatory component. Lamb et al. reported that 24% of dogs with an inflammatory CSF have a normal brain MRI [22], which implies that some MUO patients might present an unremarkable MRI. Moreover, an influence of the immune system on seizures and epileptogenesis is intensively investigated in human medicine as this might be an underlying mechanism for pharmacoresistance [42].

The non-CNS group served as negative control, since healthy dogs were unavailable due to ethical reasons. The group comprised dogs with a full neurological work-up that did not show structural CNS changes in the MRI or CSF abnormalities such as dogs with otitis media or cranial nerve neuropathy. Although an involvement of the CNS could not be excluded completely, the absence of CSF-specific OCBs in this group reflects the findings of the previously published data on OCBs in healthy dogs [24].

There are some limitations in the current study. As already outlined above, a misdiagnosis of MUO due to lack of postmortem confirmation as well as clear guidelines for the clinical diagnosis of MUO, with special attention to CSF findings, is possible. Based on the invasive nature of a general anesthesia and an atlanto-occipital CSF puncture, a healthy control group was not available. We therefore included a non-CNS group with diseases not affecting the CNS and normal MRI and CSF examination and compared the MUO group to various categories of diseases. Nevertheless, an additional completely healthy control group would have been interesting for comparison and preferable as negative control. Moreover, a comparison between infectious meningoencephalitides versus MUO is missing in the current study. Only very few cases with confirmed infectious meningoencephalitis were diagnosed during the study period, not allowing statistical comparison. A canine positive control was not available, mainly as only small volumes of CSF are available in dogs. Because of the declared cross-reactivity of the anti-canine IgG antibody used in the study, a human sample with CSF-specific OCBs was used as positive control. One additional limitation we would like to mention is that the antibody used in the presented study was not found to be superior in a comparison of two different canine anti-IgG antibodies [29]. However, no major differences were shown concerning the overall result comparing both canine anti-IgG antibodies. Finally, assessment of OCBs in CSF and serum is a qualitative method not allowing quantification.

In conclusion, similar to human MS, dogs with MUO showed the highest prevalence of CSF-specific OCBs of the examined disease categories although the current study could not prove whether one of the MUO subtypes might show comparably high CSF-specific OCB prevalence as in MS due to insufficient numbers of cases with histopathological information. This finding indicates an inflammatory B cell response and gives further insights in the disease pathogenesis in MUOs. So far, inflammatory cells in brains of dogs with GME were mainly characterized as T cells, suggesting a T cell-mediated delayed-type hypersensitivity, whereas B cells were only randomly identified [3]. Regarding the results of the current study an involvement of B cells in MUO seems likely, but needs to be further investigated. Additional studies would be needed to further characterize the prevalence of CSF-specific OCBs of the MUO subtypes as well as to evaluate whether a certain MUO subtype could serve as a potential large animal model for MS.

## Supporting information

S1 Raw image Immunoblot with serum and CSF samples from five canine patients (nos. 1–5) and one human MS patient (no. 6) after isoelectric focusing; original uncropped image. The image was scanned (Canon TS 5350a) in order to reach the best resolution possible. The loading order was from left to right, starting with dog no. 1 with the serum sample, then CSF sample etc. Dog no. 1 (study no. 82) was diagnosed with intracranial neoplasia, dog no. 2 (study no. 83) and dog no. 3 (study no. 84) with idiopathic epilepsy, dog no. 4 (study no. 85) with MUO, and dog no. 5 (study no. 86) with a disease outside the CNS. The samples from the human MS patient (study no. 23/2020) served as positive controls. CSF-specific OCBs were detected in dog no. 4 (study no. 85). Fig 5 in the manuscript was generated from this original image. CSF-specific OCBs (equivalent to pattern 2 and 3 of the described patterns in human medicine) were highlighted between arrowheads in the adjusted Fig 5. CSF = cerebrospinal fluid, MS = multiple sclerosis, MUO = meningoencephalitis of unknown origin, CNS = central nervous system, OCBs = oligoclonal bands.
(PDF)

S1 Table Minimal data set. Data used to reach the conclusions drawn in the manuscript.
(DOCX)

S2 Table Minimal data set containing anonymized study data. M = MUO, meningoencephalitis of unknown origin, E = idiopathic epilepsy, N = intracranial neoplasia, S = steroid-responsive meningitis-arteritis, D = intervertebral disc disease, C = non-CNS disease, TNCC = total nucleated cell count.
(XLSX)
