# Supplementary material for: Assessment of oligoclonal bands in cerebrospinal fluid and serum of dogs with meningoencephalitis of unknown origin
Source: PLoS One. 2023 Jan 25;18(1):e0280864. doi: 10.1371/journal.pone.0280864 (PMC9876372; doi:10.1371/journal.pone.0280864)
Supplement: S1 Raw image — The image was scanned (Canon TS 5350a) in order to reach the best resolution possible. The loading order was from left to right, starting with dog no. 1 with the serum sample, then CSF sample etc. Dog no. 1 (study no. 82) was diagnosed with intracranial neoplasia, dog no. 2 (study no. 83) and dog no. 3 (study no. 84) with idiopathic epilepsy, dog no. 4 (study no. 85) with MUO, and dog no. 5 (study no. 86) with a disease outside the CNS. The samples from the human MS patient (study no. 23/2020) served as positive controls. CSF-specific OCBs were detected in dog no. 4 (study no. 85). Fig 5 in the manuscript was generated from this original image. CSF-specific OCBs (equivalent to pattern 2 and 3 of the described patterns in human medicine) were highlighted between arrowheads in the adjusted Fig 5. CSF = cerebrospinal fluid, MS = multiple sclerosis, MUO = meningoencephalitis of unknown origin, CNS = central nervous system, OCBs = oligoclonal bands. (PDF) [file pone.0280864.s001.pdf]

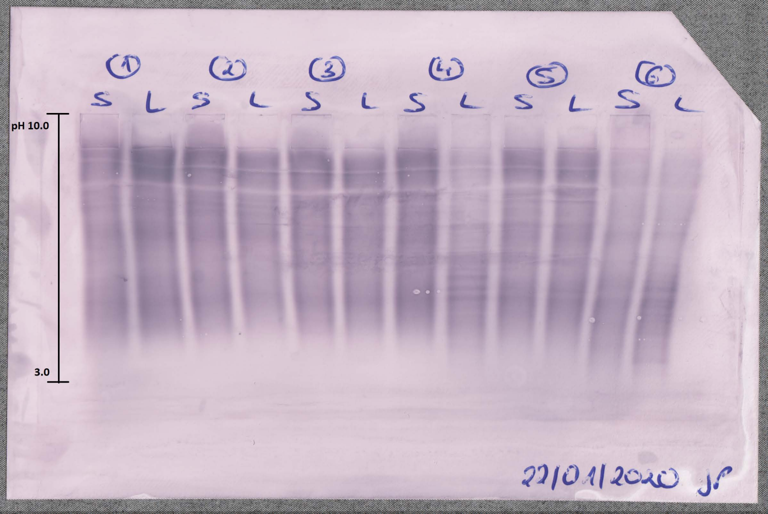

1 S L
2 S L
3 S L
4 S L
5 S L
6 S L
pH 10.0
3.0
22/01/2020
