# Supplementary material for: Assessment of oligoclonal bands in cerebrospinal fluid and serum of dogs with meningoencephalitis of unknown origin
Source: PLoS One. 2023 Jan 25;18(1):e0280864. doi: 10.1371/journal.pone.0280864 (PMC9876372; doi:10.1371/journal.pone.0280864)
Supplement: S1 Table — Data used to reach the conclusions drawn in the manuscript. (DOCX) [file pone.0280864.s002.docx]

| **Fig. #** | **Median** | **Mean** | **S.D** | **S.E** | **Statistical method used** | **P value** |
| --- | --- | --- | --- | --- | --- | --- |
| **Fig. 1** |  |  |  |  | Kruskal-Wallis One-way ANOVA | P < .01 |
| CSF-specific OCBs n | 39.78 | 178.60 | 434.06 | 45.20 |  |  |
| CSF-specific OCBs y | 114.39 | 237.51 | 452.00 | 84.34 |  |  |
| **Fig. 2** |  |  |  |  | Kruskal-Wallis One-way ANOVA | P < .001 |
| Marked | 345.55 | 565.68 | 652.14 | 85.47 |  |  |
| Mild | 108.5 | 279.44 | 661.31 | 85.47 |  |  |
| Missing | 650.56 | 650.54 | 674.60 | 276.95 |  |  |
| Normal | 38.07 | 53.93 | 56.32 | 44.63 |  |  |
|  |  |  |  |  |  |  |
|  | **Normal** | **Mononuclear** | **Neutrophilic** | **Mixed** |  |  |
| **Fig. 3** |  |  |  |  | Logistic regression and contingency tables: Pearson’s Chi-Square | P < .001 |
| CSF-specific OCBs n in % | 84.42 | 41.18 | 85.71 | 83.33 |  |  |
| CSF-specific OCBs y in % | 15.58 | 58.82 | 14.29 | 16.67 |  |  |
|  |  |  |  |  |  |  |
|  | **Normal** | **Mild** | **Marked** |  |  |  |
| **Fig. 4** |  |  |  |  | Logistic regression and contingency tables: Pearson’s Chi-Square | P > .1 |
| CSF-specific OCBs n in % | 83.12 | 61.90 | 76.19 |  |  |  |
| CSF-specific OCBs y in % | 16.88 | 38.10 | 23.81 |  |  |  |
